# Thymoquinone and caffeic acid phenethyl ester mitigate experimental ulcerative colitis: a biochemical and histopathological study

**DOI:** 10.1590/acb412226

**Published:** 2026-04-17

**Authors:** Can Ersak, Ender Ergüder, Jafar Nouri Nojadeh, Saygin Altiner, Mevlut Recep Pekcici

**Affiliations:** 1 Health Sciences University – Ankara Training and Research Hospital – General Surgery – Ankara – Turkey.; 2 Kizilcahamam State Hospital – General Surgery Department – Ankara – Turkey.; 3 Health Sciences University – Etlik City Hospital – General Surgery Department – Ankara – Turkey.; 4 Ankara University – Faculty of Medicine – Department of Medical Biochemistry – Ankara – Turkey.; 5 Gazi University – Faculty of Medicine – Department of General Surgery – Ankara – Turkey.

**Keywords:** Colitis, Ulcerative, Oxidative Stress, Anti-Inflammatory Agents

## Abstract

**Purpose:**

To establish an acetic acid-induced acute ulcerative colitis model in rats and to evaluate the clinical, biochemical, and histopathological effects of thymoquinone (TQ) and caffeic acid phenethyl ester (CAPE) in this model.

**Methods:**

Forty male Wistar albino rats (190–303 g) were randomly assigned to five groups (n = 8). All groups except the control received rectal acetic acid (4%) to induce colitis. Group 1 (control) received intraperitoneally (IP) 0.9% saline for five days. Group 2 (colitis) received IP 10% ethanol (vehicle for CAPE). Group 3 (TQ) received IP thymoquinone (10 mg/kg/day). Group 4 (CAPE) received IP caffeic acid phenethyl ester (10 µmol/kg/day). Group 5 (TQ+CAPE) received both agents at the same doses. On day 5, animals were euthanized, and colon and serum samples were collected for biochemical and histopathological analysis.

**Results:**

Significant differences were observed in the disease activity index, tumor necrosis factor-α, catalase, glutathione peroxidase, interleukin-10, and malondialdehyte indicating a balance between oxidant and antioxidant parameters. CAPE was found to have a greater anti-inflammatory effect than TQ histopathologically. The combined administration of CAPE and TQ mitigated oxidative stress as demonstrated by decreased oxidative stress biomarkers and improved antioxidant protection.

**Conclusion:**

Both CAPE and TQ demonstrated anti-inflammatory and antioxidant effects in an acetic acid-induced colitis model, with CAPE showing greater histological improvement. Their combined administration yielded additional benefits in oxidative stress reduction. These findings suggest that CAPE and TQ, particularly in combination, may hold promise as adjunctive agents in colitis management and warrant further investigation.

## Introduction

Thymoquinone (TQ), the most abundant component of Nigella sativa (black cumin) seed oil, is attributed with many of the plant’s therapeutic properties. TQ has been studied for its wide range of pharmacological effects, including antioxidant, and anti-inflammatory activities. The anti-inflammatory effects of TQ are caused by the suppression of nuclear factor-kappa B (NF-κB) activity and subsequent decrease in pro-inflammatory cytokine levels including tumor necrosis factor-alpha (TNF-α)^1,2^. It is also known for its gastroprotective and hepatoprotective properties. Literature suggests that TQ has a low side effect profile with no significant toxicity^3^. These properties make TQ a promising candidate for treating inflammatory diseases like ulcerative colitis (UC).

Caffeic acid phenethyl ester (CAPE) is a natural bioactive compound, commonly extracted from propolis, a bee product. It has demonstrated diverse pharmacological potentials, including anticancer, anti-inflammatory, antioxidant, antibacterial, antifungal, and protective effects on the nervous system and multiple organs since it was found to be a potent NF-κB inhibitor^4,5^.

UC is a chronic inflammatory bowel disease characterized by inflammation and ulceration of the colon and rectum. The incidence of UC is increasing worldwide^6,7^. While current treatment options include anti-inflammatory drugs, immunosuppressive drugs, biologic agents, and lifestyle changes, UC remains a chronic disease^8^.

Although both TQ and CAPE have individually demonstrated anti-inflammatory and antioxidant activity in experimental colitis, direct head-to-head comparative studies of these agents in the same model are scarce. Furthermore, to our knowledge, this is the first study to assess the additive effect of co-administering TQ and CAPE at standardized doses in an acute acetic acid-induced colitis model. Unlike studies using propolis—a complex mixture in which CAPE is only one component—, our work isolates the pharmacologic impact of CAPE and TQ alone and in combination, enabling a clearer understanding of their individual and combined therapeutic potentials.

In this study, acetic acid-induced colitis was used to model acute colitis in rats, and disease progression was monitored by the disease activity index (DAI). The objective was to investigate the clinical, biochemical, and histopathological effects of TQ and CAPE on colitis.

## Methods

This study was conducted at the Experimental Animal Research Laboratory, Ankara Training and Research Hospital, University of Health Sciences, Ankara, Turkey. The experimental protocol was reviewed and approved by the Animal Experiments Local Ethics Committee of the Ankara Training and Research Hospital, University of Health Sciences (Approval No: 23/09, Date: March 28, 2023). All experimental procedures complied with the European Convention for the Protection of Vertebrate Animals Used for Experimental and Other Scientific Purposes. The number of animals used was based on *a priori* power analysis (G*Power 3.1), targeting a medium effect size with 80% power at a significance level of 0.05, resulting in a total sample size of 40 rats. The design adhered to the principles of the three Rs (replacement, reduction, and refinement).

Forty male Wistar albino rats (190–303 g, mean = 247.1 g) were obtained from Saki Yenili Experimental Animals Research Center. All animals included in the study were male Wistar Albino rats, aged 8–10 weeks old, weighing between 190 and 303 grams at the start of the experiment. Rats were obtained from a certified breeding facility (Saki Yenili Experimental Animals Center) and were clinically healthy, showing no signs of infection, distress, or prior exposure to experimental procedures. Inclusion criteria required that all animals had comparable age and weight range, and were free of visible abnormalities or disease. No animals were excluded from the study after group allocation.

All animals were housed under standard laboratory conditions (22 ± 2°C, 12-hour light/dark cycle) and had *ad libitum* access to standard rodent chow and water throughout the study period.

The study was conducted at the Hüsnü Sakal Experimental and Clinical Research Center (University of Health Sciences, Ankara, Turkey). Rats were randomly assigned to five groups (n = 8 per group) based on body weight. DAI evaluations and histological assessments were performed by independent researchers blinded to the group allocations.

### Pharmacological agents

Acetic acid (CH3COOH) (Glacial 99–100%) was obtained from Tekkim Chemical Industry and Trade Limited Company. CAPE (CAS: 331-39-5) was supplied by Merck through Sigma-Aldrich Chemie GmbH. TQ (CAS: 490-91-5) was also obtained from Merck through Sigma-Aldrich Chemie GmbH.

### Groups and protocol

A 4% acetic acid solution was administered rectally using a feeding catheter, followed by a saline enema after 5 minutes to induce colitis. Treatments were administered intraperitoneally (IP) once daily for five days as follows:

- Group 1 (control): 1 mL IP 0.9% saline;
- Group 2 (colitis): IP 10% ethanol (vehicle control for CAPE);
- Group 3 (TQ): TQ 10 mg/kg/day, dissolved in 0.9% saline;
- Group 4 (CAPE): CAPE 10 µmol/kg/day, dissolved in 10% ethanol;
- Group 5 (TQ+CAPE): both agents administered at the above doses, each in its respective vehicle.

On day 5, animals were anesthetized and euthanized. Whole colon segments were dissected and collected for biochemical and histopathological analyses. Blood samples were obtained via cardiac puncture for serum biochemical assays.

An independent researcher, blinded to group details, assessed the subjects daily for weight loss, stool consistency, and rectal bleeding. Weight loss was scored based on percentage loss: 0 for no loss, 1 for 1–5%, 2 for 5–10%, 3 for 10–15%, and 4 for more than 15%. Stool consistency was evaluated, with 0 indicating normal stools, 2 for loose stools that did not adhere to the anus, and 4 for diarrhea adhering to the anus. Rectal bleeding was scored as 0 for no bleeding, 2 for occult blood positive, and 4 for visible hemorrhage adhering to the anus. Occult blood in the stool was detected using the Gikan card test and evaluated by the Guaiac method.

### Biochemical analysis

Tissue samples were frozen at -80°C until analysis. For tissue homogenization, samples were mixed with saline (1 g tissue/5 mL SF) and processed using a bullet blender homogenizer. Homogenates were centrifuged at 5,000 rpm for 20 minutes at 4°C, and supernatants were collected for biochemical assays. Blood samples were centrifuged at 3,500 rpm for 5 minutes at 4°C, and serum was stored at -80°C. Interleukin (IL)-10 and TNF-alpha levels were measured using enzyme-linked immunosorbent assay (ELISA) kits (Cloud-Clone Corp) following the manufacturer’s protocol. Caspase-3 was also quantified using a sandwich ELISA method. Additional biochemical parameters such as malondialdehyde (MDA), catalase (CAT), glutathione peroxydase (GSH-Px), superoxide dysmutase (SOD), xantine oxidase (XO) and nitric oxide (NO) were measured spectrophotometrically at 532, 240, 340, 560, 290, and 540 nm, respectively.

### Histopathology assessment

Total colon samples were fixed in formalin, embedded in paraffin, and sectioned at 4-µm thickness. Hematoxylin-eosin (HE) staining was performed, and the samples were examined using a ZEISS light microscope. Histological colitis damage scores were evaluated based on Dieleman’s scoring system, assessing inflammation severity, extent, and crypt damage. Inflammation was scored from 0 to 3, crypt damage from 0 to 4, with percentage involvement recorded.

The data were analyzed using IBM Statistical Package for the Social Sciences V23. The normality of the data distribution was assessed using the Shapiro-Wilk’s test. For comparisons of normally distributed data across three or more groups, one-way analysis of variance (ANOVA) was employed, with post-hoc analyses conducted using Tukey’s test. For non-normally distributed data across three or more groups, the Kruskal-Wallis’ H test was used, followed by multiple comparisons with Dunn’s test. Categorical data were compared using the Fisher-Freeman-Halton’s test. Results were presented as frequency (percentage) for categorical variables, and as mean ± standard deviation or median (minimum-maximum) for quantitative variables. A significance level of p < 0.050 was considered statistically significant.

## Results

### Disease activity index

Comparative analysis of the DAI revealed significant differences across all components. Median weight loss scores differed significantly among the groups (p = 0.003), with the control group scoring 0 and all other groups scoring between 1 and 1.5. Stool consistency and rectal bleeding scores also varied significantly (*p* = 0.005 and *p* = 0.027, respectively). The colitis (ethanol) group exhibited the highest cumulative scores for stool consistency (8) and rectal bleeding (7), indicating severe clinical symptoms. Treatment groups, particularly the combination group, showed notable improvement in these parameters.

Total DAI scores differed significantly among groups (*p* = 0.001), with median scores of 0 (control), 16.5 (ethanol), 10 (TQ), 9 (CAPE), and 9.5 (CAPE+TQ) (Table 1).

**Table 1:** Disease activity index scores across the groups.

	Saline (Control)	Ethanol-Colitis	TQ-colitis	CAPE-colitis	CAPE+TQ-colitis	Total	Test statistics	*p* -value*
Weight score	0 ± 0	1.5 ± 0.76	1 ± 0.53	1.5 ± 1.69	1.5 ± 0.93	1.1 ± 1.08	16.299	0.003
	0 (0–0)^b^	1 (1–3)^a^	1 (0–2)^ab^	1 (0–4)^ab^	1.5 (0–3)^a^	1 (0–4)		
Consistency score	1 ± 1.51	8.25 ± 3.28	5.25 ± 5.23	5.5 ± 4.63	5 ± 2.39	5 ± 4.2	14.774	0.005
	0 (0–4)^b^	8 (6–16)^a^	4 (0–14)^ab^	4 (0–12)^ab^	5 (2–8)^ab^	4 (0–16)		
Bleeding score	0 ± 0	6.75 ± 4.4	3.75 ± 4.71	4.5 ± 5.1	4.75 ± 5.23	3.95 ± 4.7	10.977	0.027
	0 (0–0)^b^	7 (0–14)^a^	1 (0–10)^ab^	3 (0–14)^ab^	3 (0–12)^ab^	2 (0–14)		
DAI total	1 ± 1.51	16.5 ± 5.29	10 ± 8.3	11.5 ± 8.82	11.25 ± 7.48	10.05 ± 8.24	19.169	0.001
	0 (0–4)^b^	16.5 (9–23)^a^	10 (0–26)^ab^	9 (3–30)^a^	9.5 (3–22)^a^	8.5 (0–30)		

DAI: disease activity index; TQ: thymoquinone; CAPE: caffeic acid phenethyl ester. Source: Elaborated by the authors.

### Tissue and serum biochemical parameters

Tissue IL-10 levels differed significantly between groups (p = 0.002), with the control group showing a median of 141.14, while values in the ethanol, TQ, CAPE, and combination groups were 137.79, 76.01, 80.32, and 78.17, respectively (Table 2). Catalase (CAT) activity was significantly higher in the TQ, CAPE, and TQ+CAPE groups compared to the colitis group (*p* = 0.001). GSH-Px levels also showed significant differences among groups (*p* = 0.003).

**Table 2:** Comparison of quantitative parameters in tissue by groups.

	Group					Total	Test statistics	*p* -value*
	Saline (Control)	Ethanol-Colitis	TQ-colitis	CAPE-colitis	CAPE+TQ-colitis			
TNF-alfa	60.7 ± 17.91	41.96 ± 26.29	45.43 ± 13.86	44.21 ± 15.89	43.54 ± 9.93	47.17 ± 18.09	4.466	0.347**
	55.75 (43.27–93)	50.57 (11.2–69.57)	45.29 (26.69–67.86)	43.62 (14.7–64.92)	42.73 (32.38–63.15)	46.6 (11.2–93)		
IL-10	143.67 ± 43.77	153.53 ± 47.9	81.01 ± 41.16	88.21 ± 36.51	81.62 ± 32.97	109.61 ± 50.5	17.405	0.002**
	141.14 (95.09–231.22)^a b^	137.79 (106.52–259.2)^b^	76.01 (32.92–157.32)^a^	80.32 (43.22–134.81)^a b^	78.17 (37.24–129.63)^a^	106.45 (32.92–259.2)		
Caspase3	1.05 ± 0.54	1.09 ± 0.72	1.16 ± 0.47	0.87 ± 0.36	0.8 ± 0.37	1 ± 0.5	3.407	0.492**
	0.77 (0.6–2.16)	0.84 (0.44–2.75)	1.09 (0.78–2.21)	0.93 (0.12–1.39)	0.78 (0.07–1.31)	0.85 (0.07–2.75)		
MDA	16.37 ± 0.67	15.96 ± 2.04	14.55 ± 4.3	16.38 ± 1.48	15.97 ± 0.96	15.85 ± 2.28	0.535	0.712*
	16.5 (15.48–17.1)	16.29 (12.98–18.31)	15.61 (6.37–19.84)	15.89 (14.92–18.79)	15.69 (14.6–17.34)	15.93 (6.37–19.84)		
CAT	117.66 ± 39.51^b c^	101 ± 28.1^b^	158.5 ± 41.03^a c^	154.09 ± 38.92^a c^	172.56 ± 13.35^a^	140.76 ± 42.07	6.318	0.001*
	118.55 (58.6–197.6)	106.85 (51.2–130.3)	150.8 (115.7–224)	147.85 (106.9–213.7)	173.5 (152.3–193.2)	128.85 (51.2–224)		
GSH-Px	0.36 ± 0.01	0.4 ± 0.04	0.36 ± 0.03	0.43 ± 0.05	0.41 ± 0.05	0.39 ± 0.05	16.161	0.003**
	0.35 (0.34–0.39)^b^	0.39 (0.35–0.49)^a b^	0.36 (0.29–0.4)^a b^	0.43 (0.36–0.5)^a^	0.43 (0.35–0.47)^a b^	0.38 (0.29–0.5)		
SOD	105.14 ± 15.32	77.9 ± 30.5	107.93 ± 12.94	110.08 ± 37.4	97.96 ± 7.4	99.8 ± 25.3	8.007	0.091**
	106.1 (84.8–129.3)	78.95 (28.2–122.4)	113.15 (89.5–123)	102.5 (78.8–200)	95.9 (90.3–109.8)	99.9 (28.2–200)		
XO	0.02 ± 0.03	0 ± 0	0.01 ± 0.03	0.03 ± 0.08	0 ± 0	0.01 ± 0.04	4.283	0.369**
	0 (0–0.07)	0 (0–0)	0 (0–0.07)	0 (0–0.22)	0 (0–0.01)	0 (0–0.22)		
NO	106.31 ± 76.21	70.47 ± 50.12	62.59 ± 34.97	109.25 ± 42.92	69.94 ± 16.27	83.71 ± 49.96	5.206	0.267**
	91.75 (25–268)	58.88 (10–167.25)	56.63 (6.5–113.75)	100.63 (53.75–169.75)	62.38 (53–91)	76.5 (6.5–268)		
NOS	11.78 ± 1.76	10.83 ± 2.05	11.96 ± 1.53	11.78 ± 1.48	10.61 ± 1.64	11.39 ± 1.71	1.061	0.390*
	12.32 (8.96–13.69)	11.33 (6.49–13.24)	12.1 (9.56–13.99)	11.29 (9.79–13.76)	10.26 (8.96–14.06)	11.38 (6.49–14.06)		

TQ: thymoquinone; CAPE: caffeic acid phenethyl ester; TNF: tumor necrosis factor; IL: interleukin; MDA: malondialdehyde; CAT: catalase; GSH-Px: glutathione peroxidase; SOD: superoxide dismutase; XO: xanthine oxidase; NO: nitric oxide; NOS: nitric oxide synthase;

* one-way analysis of variance test; *Kruskal-Wallis’ H test;

a no significant difference between groups with the same letter;

b mean ± standard deviation;

c median (minimum–maximum).

Source: Elaborated by the authors.

Serum TNF-α levels were significantly different (*p* = 0.002), with lower levels in the CAPE and combination groups compared to both the colitis and control groups. Significant intergroup differences were also observed in serum levels of caspase-3, MDA, and CAT. NOS levels varied significantly across groups (*p* = 0.013), but multiple comparisons did not reveal significant differences between specific pairs (Table 3).

**Table 3:** Comparison of quantitative parameters in serum by group.

	Group					Total	Test statistics	*p* -value*
	Saline (Control)	Ethanol-Colitis	TQ-colitis	CAPE-colitis	CAPE+TQ-colitis			
TNF alfa	100.16 ± 21.99b	71.18 ± 18.15ab	79.07 ± 27.19ab	61.51 ± 14.57a	53.37 ± 24.28a	73.06 ± 26.23	5.501	0.002*
	102.41 (54.84–129.86)	75.39 (36.36–88.71)	81.51 (41.53–127.1)	59.77 (43.21–91.65)	46.82 (32.51–96.01)	70.47 (32.51–129.86)		
Caspase3	1.14 ± 0.17	1.49 ± 0.44	1.55 ± 0.64	1.81 ± 0.54	2.44 ± 1.08	1.69 ± 0.75	14.497	0.006**
	1.13 (0.86–1.38)^b^	1.31 (1.2–2.47)^a b^	1.47 (1.02–3.02)^a b^	1.69 (1.06–2.66)^a b^	2.27 (1.05–4.17)^a^	1.45 (0.86–4.17)		
MDA	16.32 ± 1.06	12.28 ± 3.99	12.02 ± 3.68	14.8 ± 1.31	11.47 ± 4.05	13.38 ± 3.51	15.349	0.004**
	16.49 (14.52–17.82)^b^	13.95 (5.65–15.57)^a b^	12.83 (6.53–17.42)^a^	14.8 (12.58–16.78)^ab^	11.17 (3.15–16.21)^a^	14.28 (3.15–17.82)		
CAT	2.76 ± 2.11	2.76 ± 1.64	4.4 ± 2.71	10.06 ± 9.73	13.9 ± 3.33	6.78 ± 6.48	20.407	< 0.001**
	1.5 (1.5–7.3)^b^	2.2 (1.5–5.9)^b^	3.65 (1.5–8.8)^a b^	5.85 (1.5–26.4)^a b^	13.2 (10.2–19)^a^	4.4 (1.5–26.4)		
GSH-Px	0.16 ± 0.02	0.17 ± 0.02	0.18 ± 0.03	0.16 ± 0.05	0.14 ± 0.06	0.16 ± 0.04	1.441	0.241*
	0.16 (0.13–0.18)	0.17 (0.12–0.19)	0.18 (0.15–0.25)	0.17 (0.08–0.24)	0.16 (0–0.19)	0.16 (0–0.25)		
SOD	60.95 ± 10.63	67.41 ± 10.86	121.21 ± 157.28	59.39 ± 13.54	60.85 ± 6.54	73.96 ± 71.43	3.894	0.421**
	62.8 (43.3–75)	69.25 (50.4–78.2)	66.95 (53.2–509.7)	64.55 (31.2–70.8)	61.15 (50.2–68.7)	65.2 (31.2–509.7)		
XO	0.09 ± 0.03	0.07 ± 0.04	0.06 ± 0.02	0.07 ± 0.04	0.06 ± 0.04	0.07 ± 0.04	4.16	0.385**
	0.09 (0.02–0.14)	0.09 (0–0.11)	0.06 (0.04–0.1)	0.08 (0–0.11)	0.05 (0–0.12)	0.08 (0–0.14)		
NO	18.72 ± 11.01	19.16 ± 5.85	21.78 ± 11.78	20.56 ± 9.19	23.18 ± 11.81	20.62 ± 9.72	1.337	0.855**
	17.38 (10.25–44)	19.88 (11.75–30)	16.75 (11.75–44)	20.75 (7.25–33.5)	18.5 (12.5–44.25)	18.5 (7.25–44.25)		
NOS	9.45 ± 3.75	9.41 ± 2.2	9.49 ± 1.99	16.3 ± 6.45	17.77 ± 8.31	12.35 ± 6.06	12.686	0.013**
	7.65 (6.98–17.55)	9.23 (7.16–14.06)	9.81 (6.6–12.11)	15.51 (7.54–27.64)	20.66 (4.5–28.01)	10.24 (4.5–28.01)		

TQ: thymoquinone; CAPE: caffeic acid phenethyl ester; TNF: tumor necrosis factor; IL: interleukin; MDA: malondialdehyde; CAT: catalase; GSH-Px: glutathione peroxidase; SOD: superoxide dismutase; XO: xanthine oxidase; NO: nitric oxide; NOS: nitric oxide synthase;

* one-way analysis of variance test; *Kruskal-Wallis’ H test;

a no significant difference between groups with the same letter;

b mean ± standard deviation; cmedian (minimum–maximum).

Source: Elaborated by the authors.

### Histopathological evaluation

**Figure 1:**
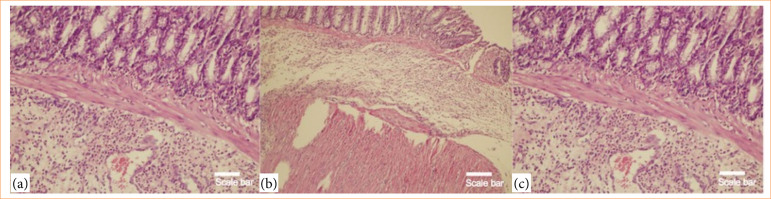
Representative histopathological images from the colitis group (hematoxylin and eosin staining). (a) Low-power view (lowest magnification) showing severe full-thickness mucosal and submucosal ulceration with dense inflammatory cell infiltration (histological grade 3); (b) low-power view (10× objective) demonstrating diffuse transmural inflammatory infiltration and marked edema; (c) high-power view (20× objective) showing crypt destruction, cryptitis, and dense inflammatory infiltration in the mucosa and submucosa. Scale bars are shown for reference purposes only; original magnifications are indicated in each panel.

Source: Elaborated by the authors.

**Figure 2:**
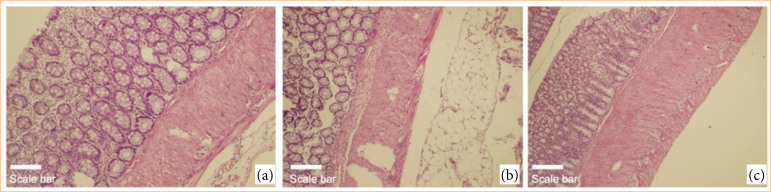
Representative histopathological images from the treatment groups (hematoxylin and eosin staining). (a) Normal colonic mucosal architecture in the control group; (b) low-power view (10× objective) showing mild full-thickness inflammatory infiltration in the caffeic acid phenethyl ester (CAPE)-treated group; (c) high-power view (20× objective) demonstrating minimal inflammatory changes limited to the mucosal layer in the CAPE+thymoquinone group. Scale bars are shown for reference purposes only; original magnifications are indicated in each panel.

Source: Elaborated by the authors.

Histopathological analysis demonstrated statistically significant differences among groups in terms of inflammation severity (*p* = 0.011), distribution (*p* = 0.016), crypt damage (*p* = 0.025), and percentage of colonic involvement (*p* = 0.017). The control group exhibited minimal or no inflammation, while the ethanol and treatment groups displayed varying degrees of mild to severe mucosal and transmural involvement. However, post-hoc comparisons did not reveal statistically significant pairwise differences for these categorical parameters (Figs. 1 and 2).

Total histopathological scores differed significantly between groups (*p* = 0.004). The median total score was 0 in the control group, 7.5 in the ethanol group, 6.5 in the TQ group, 4 in the CAPE group, and 5 in the CAPE+TQ group. Significant differences were observed between the control group and both the ethanol and TQ groups (Tables 4 and 5).

**Table 4:** Comparison of categorical pathological scores by group.

	Group					Test statistics	*p* -value*
	Saline (control)	ETH	TQ	CAPE	CAPE+TQ		
Inflammation score							
None	5 (62.5)	0 (0)	0 (0)	0 (0)	0 (0)	20.497	0.011
Mild	3 (37.5)	3 (37.5)	3 (37.5)	6 (75)	5 (62.5)		
Moderate	0 (0)	3 (37.5)	4 (50)	2 (25)	1 (12.5)		
Severe	0 (0)	2 (25)	1 (12.5)	0 (0)	2 (25)		
Spread of inflammation							
None	5 (62.5)	0 (0)	0 (0)	0 (0)	0 (0)	19.824	0.016
Mucosa	3 (37.5)	3 (37.5)	5 (62.5)	6 (75)	3 (37.5)		
Submucosa	0 (0)	2 (25)	2 (25)	1 (12.5)	4 (50)		
Transmural	0 (0)	3 (37.5)	1 (12.5)	1 (12.5)	1 (12.5)		
Crypt damage							
No damage	5 (62.5)	0 (0)	0 (0)	0 (0)	0 (0)	21.78	0.025
Basal 1/3 damaged	2 (25)	4 (50)	4 (50)	6 (75)	6 (75)		
Basal 2/3 damaged	1 (12.5)	3 (37.5)	3 (37.5)	2 (25)	1 (12.5)		
Crypt	0 (0)	0 (0)	0 (0)	0 (0)	1 (12.5)		
Loss of crypt and surface epithelium	0 (0)	1 (12.5)	1 (12.5)	0 (0)	0 (0)		
Percentage involvement (%)							
0	5 (62.5)	0 (0)	0 (0)	0 (0)	0 (0)	22.767	0.017
1–25	3 (37.5)	2 (25)	3 (37.5)	6 (75)	6 (75)		
25–50	0 (0)	3 (37.5)	3 (37.5)	1 (12.5)	1 (12.5)		
51–75	0 (0)	2 (25)	1 (12.5)	1 (12.5)	1 (12.5)		
76–100	0 (0)	1 (12.5)	1 (12.5)	0 (0)	0 (0)		

TQ: thymoquinone; CAPE: caffeic acid phenethyl ester; ETH: ethanol. Source: Elaborated by the authors.

**Table 5:** Comparison of total scores by group.

	Total		Test statistics	*p* -value*
	Mean ± standard deviation	Median (min–max)		
Group				
Saline (control)	1.63 ± 2.26	0 (0–5)^b^	15.606	**0.004**
ETH	7.88 ± 3.44	7.5 (4–14)^a^		
TQ	7 ± 3.16	6.5 (4–14)^a^		
CAPE	5.25 ± 2.31	4 (4–9)^ab^		
CAPE+TQ	6.13 ± 2.8	5 (4–11)^ab^		
Total	5.58 ± 3.46	4.5 (0–14)		

* Kruskal-Wallis H test;

a-b there is no significant difference between groups with the same letter;

TQ: thymoquinone; CAPE: caffeic acid phenethyl ester; ETH: ethanol.

## Discussion

The DAI was used to evaluate colitis activity and symptoms, a scoring system first validated by Murthy et al.^9^. Daily DAI assessments were performed by an independent researcher, and the ethanol-colitis group had the highest median score (16.5), indicating the most severe colitis symptoms. The high DAI scores in the ethanol-colitis models compared to the saline group confirmed the appropriate induction of colitis. Although standardized macroscopic photographs of the colonic specimens were not obtained, the successful induction of colitis was objectively confirmed using the DAI, which demonstrated significantly higher clinical scores in the ethanol-colitis group compared to the other experimental groups.

Macroscopic documentation was intentionally not performed to minimize the time between euthanasia and tissue processing for biochemical analyses, as prolonged handling and delayed processing could potentially affect the levels of biomarkers with short tissue half-lives. This approach was adopted to preserve the biochemical integrity of tissue supernatants and to ensure the reliability of cytokine and oxidative stress measurements. The total DAI scores in the TQ, CAPE, and CAPE+TQ groups were significantly lower than those in the ethanol-colitis group, and the differences were statistically significant. Similar differences were observed across weight, consistency, and bleeding scores among the groups. The significant results of the DAI scores indicate that the colitis model induced in the subjects was successful. To minimize confounding effects of the different vehicles used, each compound’s vehicle was also administered to its respective control group. Group 2 received IP 10% ethanol (CAPE’s vehicle), while group 1 received IP saline (TQ’s vehicle), ensuring that solvent effects were accounted for.

This study employed an acute acetic acid-induced colitis model, which primarily mimics chemical injury rather than chronic immune-mediated inflammation. While this model is widely used to assess short-term anti-inflammatory effects, it may not fully replicate the chronic and relapsing nature of UC in humans. Therefore, the findings should be interpreted within the limitations of this acute setting, and future studies should consider using chronic colitis models for broader translational relevance.

Tissue TNF-alpha levels were the lowest in the CAPE+TQ group, while the ethanol-colitis group had the highest quantitative TNF-alpha levels, although the differences between groups were not statistically significant. In serum, the differences in TNF-alpha levels were statistically significant (*p* = 0.002), with the lowest levels observed in the CAPE and CAPE+TQ groups, which were significantly lower than in the ethanol-colitis group. This suggests a potential increase in anti-inflammatory effect when TQ and CAPE are used together. Currently, anti-TNF drugs are used in the treatment of UC, offering an alternative for steroid-resistant cases, while effectively preventing relapses and reducing colectomy rates. In this context, further clinical studies on the antioxidant properties of TQ and CAPE molecules could be beneficial for improving UC prognosis^10,11^.

Regarding tissue IL-10 levels, the ethanol-colitis group had a median of 137.79, compared to 76.01 in the TQ group, 80.32 in the CAPE group, and 78.17 in the CAPE+TQ group. The differences between the ethanol-colitis group and the TQ, CAPE, and CAPE+TQ groups were statistically significant (p = 0.002). The high IL-10 levels in colitis-induced rats are consistent with the findings in the literature and suggest a response to high cytokine secretion. In our study, the elevated IL-10 levels observed in the colitis-induced subjects align with findings in the literature. A study by Fonseca-Camarillo et al.^12^ reported higher IL-10 producing cells in UC patients compared to healthy individuals. IL-10 was concentrated in macrophages and lymphocytes and spread across a wide area from the serosa to the mucosal layer. All biopsies showed increased IL-10-reactive cells in the colon serosa. Melgar et al.^13^ also demonstrated increased IL-10 mRNA levels and IL-10 positive cells in UC patients’ T-lymphocytes. These findings support that the elevated IL-10 levels in our study reflect an inflammatory response consistent with the pathophysiology of UC.

Tissue CAT levels also showed significant differences (*p* = 0.001), with the CAPE+TQ group having the highest levels, suggesting an increased effect in enhancing antioxidant activity. Serum CAT levels were significantly different as well (*p* < 0.001), with CAPE+TQ showing the highest levels, further supporting a positive anti-inflammatory effect. In a study by Rana et al.^14^, serum CAT levels were compared between UC patients in remission and those with active colitis, showing significantly higher CAT levels in the active colitis group. CAT plays a crucial role in eliminating hydrogen peroxide, and novel agents mimicking SOD and CAT are being explored as potential treatments for UC. Shen et al. designed a nanosystem (Tpl/OxbCD NP) that effectively eliminates reactive oxygen species components by mimicking SOD and CAT functions^15^. Based on this, CAT-mimicking molecules are gaining attention in UC treatment. Our study suggests that the potential anti-inflammatory effects of TQ and CAPE may be linked to increased endogenous CAT levels. The observed difference in CAT levels between the SF group and the CAPE+TQ group suggests that the treatment may enhance CAT activity through its antioxidant effects.

For GSH-Px, statistically significant differences in tissue levels were found (*p* = 0.003), with higher levels in the CAPE and CAPE+TQ groups compared to the SF group. However, serum GSH-Px levels did not show significant differences. Tissue and serum SOD levels were not statistically significant across groups, although CAPE and TQ groups had higher quantitative levels. In a study by Okutan et al.^16^ on diabetic rat hearts, CAPE administration was examined for its effects on the oxidative/antioxidative balance. While GSH-Px activity did not differ between diabetic rats and controls, it was significantly higher in CAPE-treated subjects. This suggests, consistent with our study, that CAPE may positively influence GSH-Px activity, regardless the underlying pathological condition.

Serum caspase-3 levels showed statistically significant differences (*p* = 0.006), with the highest levels in the CAPE+TQ group, suggesting a possible involvement in the anti-inflammatory mechanisms of these agents. A study by Yu et al.^17^ demonstrated that apoptosis induced by visilizumab increases apoptosis in activated T-lymphocytes, reducing inflammation in the colonic lamina propria and facilitating rapid remission in ulcerative colitis. Similarly, Shteingart et al.^18^ showed that inhibiting the caspase-3 mechanism leads to defective T-lymphocyte responses, triggering inflammation. In our study, the observed increase in serum caspase-3 levels may indicate an increase in apoptosis. However, this remains speculative in the absence of direct evidence on immune cell populations. Further studies are needed to confirm whether caspase activation contributes to reduced mucosal inflammation^18^.

While our findings confirm significant modulation of pro- and anti-inflammatory cytokines, as well as oxidative stress markers, the underlying molecular pathways by which CAPE and TQ exert their effects were not directly assessed. In particular, both compounds are known to inhibit NF-κB signaling—a central pathway in colitis pathophysiology—, but NF-κB activation status was not evaluated in the present study. Similarly, although increased caspase-3 levels suggest enhanced apoptosis, we did not perform cell-type-specific analyses (*e.g.*, TUNEL assay, immunostaining) to verify whether immune cell apoptosis contributed to the observed anti-inflammatory effects. Future studies should explore these pathways using targeted molecular and immunohistochemical techniques to better elucidate the mechanisms involved.

Serum MDA levels were significantly different (*p* = 0.004), with lower levels in the TQ and CAPE+TQ groups compared to the ethanol-colitis group, indicating a protective effect of these molecules against oxidative damage. In a study by Kruidenier et al.^19^, investigating the relationship between oxidative damage and IBD at the cellular level, MDA levels were found to be significantly higher in the active inflammation group of UC patients compared to those without inflammation and the control group. This finding aligns with our study, in which the lowest serum MDA levels were observed in the CAPE+TQ group, suggesting that these two agents may have protective effects against oxidative damage to cell membranes.

There were no significant differences in XO levels between groups, consistent with previous studies suggesting XO may not be the primary source of superoxide production in colitis^20^.

Serum NOS levels showed a significant difference (*p* = 0.013), with higher levels in the CAPE+TQ group, which, combined with lower clinical and histopathological inflammation scores, suggests a protective role of NOS in colon structure. However, more detailed studies are needed to fully understand the mechanisms. A study on UC patients found that NO levels were significantly higher in the rectal dialysate of patients with active colitis^21^. However, the use of NOS inhibitors in experimental colitis models has yielded inconsistent results. Non-specific NOS inhibitors, such as NG-nitro-L-arginine methyl ester (L-NAME), reduced intestinal inflammation in some models, but in others, they either provided no protection or worsened inflammation^22,23^. In a study by McCafferty et al., rats genetically unable to produce NOS were compared with normal rats after acetic acid-induced colitis, showing more severe inflammation in the NOS-deficient group^24^. In our study, CAPE+TQ-treated subjects exhibited higher NOS levels and lower clinical and histopathological inflammation scores, suggesting a protective role of NOS and NO in maintaining colon cell structure. However, further research is needed to explore the underlying cellular mechanisms in detail.

Histopathological analysis revealed statistically significant differences in total inflammation scores among the groups (*p* = 0.004). The median score in the SF group was 0, while it was 7.5 in the ethanol-colitis group, 6.5 in the TQ group, 4 in the CAPE group, and 5 in the CAPE+TQ group. There was a significant difference between the SF group and the ethanol-colitis and TQ groups. CAPE and CAPE+TQ showed no significant difference from the SF group, indicating that both CAPE and the combination therapy reduced histological inflammation scores compared to TQ alone, although no statistically significant difference was observed between the CAPE and CAPE+TQ groups. In a study by Mahgoub^25^, rats pre-treated with 10 mg/kg oral TQ for three days prior to acetic acid-induced colitis showed lower levels of inflammation in colon specimens compared to the colitis-only group. This suggests that, in addition to its anti-inflammatory effect, TQ may offer protective benefits in colitis. Similarly, in our study, CAPE administered IP after the onset of acute colitis was found to be more effective in reducing inflammation, as observed in histopathological examinations. Tambuwala et al.^26^ also demonstrated that CAPE reduced fibrosis at the cellular level in a DSS-induced chronic colon inflammation model. Our results further confirm the anti-inflammatory efficacy of CAPE in acute colitis.

## Conclusion

This study demonstrated that both CAPE and TQ exert anti-inflammatory and antioxidant effects in an acetic acid-induced acute colitis model. Histological analysis indicated a more pronounced anti-inflammatory response with CAPE compared to TQ. The combination of CAPE and TQ resulted in improved biochemical parameters, particularly in oxidative stress and antioxidant enzyme levels, suggesting additive therapeutic potential. This is the first study to directly compare CAPE and TQ, and to evaluate their co-administration within the same experimental model. These findings may support further research into multi-target antioxidant therapies as potential adjuncts for colitis treatment.

## Data Availability

Data are available from the corresponding author on reasonable request.
